# 360. The diagnostic sensitivity of beta-D-glucan assay in patients with chronic disseminated candidiasis

**DOI:** 10.1093/ofid/ofac492.438

**Published:** 2022-12-15

**Authors:** Hyo-Ju Son

**Affiliations:** Uijeongbu Eulji Medical Center, Seoul, Kyonggi-do, Republic of Korea

## Abstract

**Background:**

Chronic disseminated candidiasis (CDC) is a specific syndrome in patients with hematologic malignancies which usually occurs after the recovery of neutrophils due to previous chemotherapy. The beta-D-glucan (BDG) assay has been proposed as an adjunct test for diagnosing invasive fungal infection. However, data on BDG assay in patients with CDC are scare. We aimed to investigate the diagnostic sensitivity of BDG assay in patients with CDC.

**Methods:**

All adult patients who were diagnosed as CDC in a tertiary hospital in Seoul, South Korea, from January 2017 to December 2019 and underwent BDG assay (Gold Mountain River Tech Development, Beijing, China) were retrospectively reviewed. CDC was defined by the demonstration of small, target-like abscesses in liver or spleen (bull’s-eye lesions) or in the brain at the time of neutrophil recovery after a prolonged phase of neutropenia. The values for BDG over 80 pg/mL was classified as positive.

**Results:**

A total of 20 patients were enrolled. The median age was 51 years (Interquartile range 39 – 64). Of these, 13 patients had acute myeloid leukemia, 3 acute lymphoid leukemia, 2 myelodysplastic syndrome, and 1 aplastic anemia. *Candida* spp. were isolated in 6 patients; 3 were *C. tropicalis*, 2 *C. glabrata*, and 1 *C. krusei*. Of the 20 patients, 10 (50%) revealed positive BDG results. The median BDG value was 174 pg/dL (IQR 137–402). More CDC patients with previous candidemia had positive BDG assay than those without candidemia, but with no statistical significance (4/5 (80%) vs. 6/15 (40%), *P* = 0.30). In the 7 patients with BDG assay-positive CDC, for whom follow-up BDG results were available, the BDG remained high in 6 patients (86%) for more than 4 weeks after adequate antifungal therapy. All 4 patients who died had a positive BDG assay, and 3 of them showed an increasing trend of BDG values during treatment.

**Figure:**
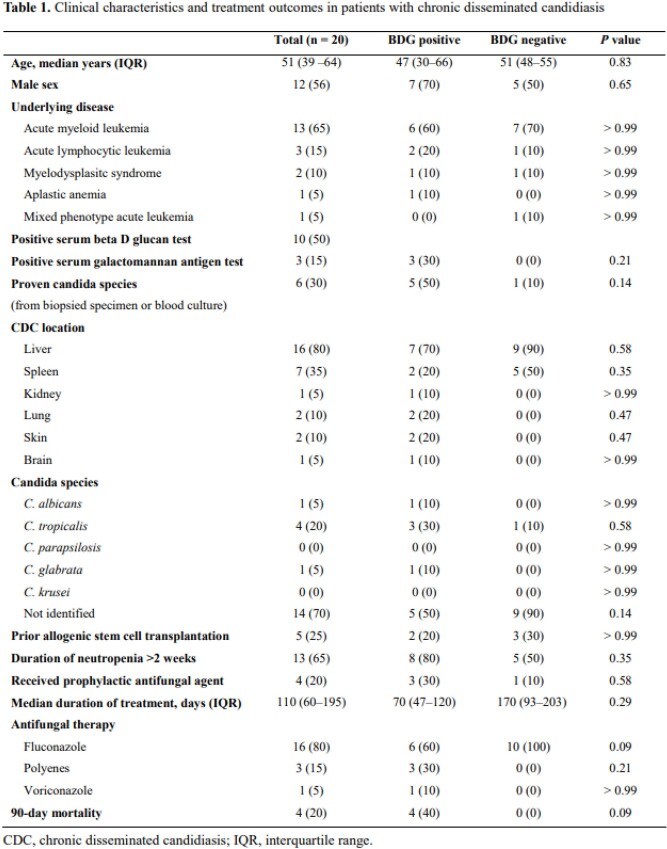

**Figure:**
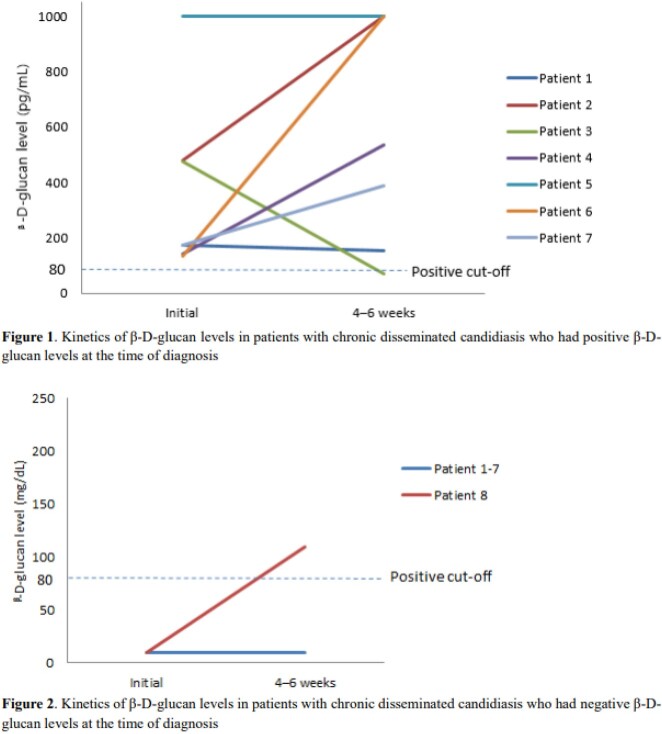

**Conclusion:**

Negative BDG assay appears to be not useful to rule out CDC. BDG assay decreased slowly during the adequate treatment of CDC.

**Disclosures:**

**All Authors**: No reported disclosures.

